# Insights into cytochrome *bc*_1_ complex binding mode of antimalarial 2-hydroxy-1,4-naphthoquinones through molecular modelling

**DOI:** 10.1590/0074-02760160417

**Published:** 2017-04

**Authors:** Ana Carolina Rennó Sodero, Bárbara Abrahim-Vieira, Pedro Henrique Monteiro Torres, Pedro Geraldo Pascutti, Célia RS Garcia, Vitor Francisco Ferreira, David Rodrigues da Rocha, Sabrina Baptista Ferreira, Floriano Paes Silva

**Affiliations:** 1 Universidade Federal do Rio de Janeiro, Faculdade de Farmácia, Laboratório de Modelagem Molecular e QSAR, Rio de Janeiro, RJ, Brasil; 2 Universidade Federal do Rio de Janeiro, Instituto de Biofísica Carlos Chagas Filho, Laboratório de Modelagem e Dinâmica Molecular, Rio de Janeiro, RJ, Brasil; 3 Instituto Nacional de Metrologia Qualidade e Tecnologia, Diretoria de Metrologia Aplicada às Ciências da Vida, Programa de Biotecnologia, Duque de Caxias, RJ, Brasil; 4 Universidade de São Paulo, Departamento de Fisiologia, São Paulo, SP, Brasil; 5 Universidade Federal Fluminense, Instituto de Química, Departamento de Química Orgânica, Niterói, RJ, Brasil; 6 Universidade Federal do Rio de Janeiro, Instituto de Química, Laboratório de Síntese Orgânica e Prospecção Biológica, Rio de Janeiro, RJ, Brasil; 7 Fundação Oswaldo Cruz-Fiocruz, Instituto Oswaldo Cruz, Laboratório de Bioquímica Experimental e Computacional de Fármacos, Rio de Janeiro, RJ, Brasil

**Keywords:** atovaquone, Plasmodium falciparum, molecular docking, surflex-dock, AutoDock4.2

## Abstract

**BACKGROUND:**

Malaria persists as a major public health problem. Atovaquone is a drug that inhibits the respiratory chain of *Plasmodium falciparum*, but with serious limitations like known resistance, low bioavailability and high plasma protein binding.

**OBJECTIVES:**

The aim of this work was to perform molecular modelling studies of 2-hydroxy-1,4-naphthoquinones analogues of atovaquone on the Q_o_ site of *P. falciparum* cytochrome *bc*_1_ complex (Pfbc_1_) to suggest structural modifications that could improve their antimalarial activity.

**METHODS:**

We have built the homology model of the cytochrome b (CYB) and Rieske iron-sulfur protein (ISP) subunits from Pfbc_1_ and performed the molecular docking of 41 2-hydroxy-1,4-naphthoquinones with known in vitro antimalarial activity and predicted to act on this target.

**FINDINGS:**

Results suggest that large hydrophobic R2 substituents may be important for filling the deep hydrophobic Q_o_ site pocket. Moreover, our analysis indicates that the H-donor 2-hydroxyl group may not be crucial for efficient binding and inhibition of Pfbc_1_ by these atovaquone analogues. The C1 carbonyl group (H-acceptor) is more frequently involved in the important hydrogen bonding interaction with His152 of the Rieske ISP subunit.

**MAIN CONCLUSIONS:**

Additional interactions involving residues such as Ile258 and residues required for efficient catalysis (e.g., Glu261) could be explored in drug design to avoid development of drug resistance by the parasite.

In 2013, it was estimated that there were almost 200 million cases of malaria and up to 550,000 deaths from this disease. The highest mortality levels are reported in Africa, where children under five years of age account for 78% of all deaths due to malaria (WHO 2014).

Human malaria is caused by infection with protozoan parasites of the genus *Plasmodium* and is transmitted by the bite of infected female mosquitoes from more than 30 anopheline species. *P. falciparum* causes the most deadly form of the disease, which predominates in Africa (WHO 2014). Despite the advances in the understanding of molecular and cell biology of *Plamodium spp*. (WHO 2014), development of new drugs to treat infections caused by these parasites is still incipient although highly needed.

The development of *P. falciparum* resistance to drugs is the main cause of reduced malaria control program performance and increasing burden. Resistance to chloroquine, one of the most widely used antimalarials, is well-known and multidrug resistance reports are becoming more common. Therefore, new strategies for its use are under constant development (Newton & White 1999, Aguiar et al. 2012, Sharma et al. 2013).

The available antimalarial drug atovaquone has potent antimalarial activity, it has low bioavailability and high plasma protein binding (Dressman & Reppas 2000). Aiming to improve bioavailability and neutralise drug resistance, several atovaquone analogs containing a naphthoquinone nucleous have been prepared. Structural changes were made mainly to the alkyl side chain, since it has been shown that the modification of this moiety alters the drug’s activity (Danoun et al. 1999) and counteracts drug resistance (Danoun et al. 1999, Sharma et al. 2013).

Atovaquone is known to inhibit the respiratory chain of the parasite and there are reports that resistance is related to mutations in the cytochrome b (CYB) gene (Perry et al. 2009). Cytochrome *bc*_1_ complex (EC 1.10.2.2, ubiquinol:cytochrome c oxido-reductase) is an essential component of the cellular respiratory chain in mitochondria (Esser et al. 2004). It is a multi-subunit membrane protein, with 10 or 11 protein chains in mitochondrial forms and three or more in bacterial complexes. Three subunits containing prosthetic groups are essential for the ET function: the CYB subunit, which has two b-type hemes (low potential bL and high potential bH heme); the CYC1 subunit, which binds a c-type heme; and the iron-sulfur protein (ISP), which possesses a 2Fe-2S cluster (Esser et al. 2004).

The three subunits important for ET function are all membrane bound. The CYB subunit is embedded in the membrane with eight TM helices, and the CYC1 and ISP each has one TM helix. While the extrinsic head domain of CYC1 is rigidly tethered to its TM helix, the connection between the ISP extrinsic domain and the TM helix is very flexible. Three distinct ISP positions in crystallographic studies of *bc*_1_ from different species have been reported: the b, c1 and intermediate position based on the locations of 2Fe-2S cluster with respect to that of Q_o_ site of CYB or c1 heme of CYC1 subunit (Esser et al. 2004).

According to the proton motive Q cycle hypothesis there are two discrete reaction sites in cytochrome *bc*_1_, a quinone reduction site near the negative side of the membrane (Q_i_ or QN) and a quinol oxidation site close to the positive side of the membrane (Q_o_ or QP), which features a bifurcated ET.

The Q_o_ pocket is highly hydrophobic and is lined with six prominent aromatic residues in addition to a number of aliphatic residues. The pocket is delimited against the membrane by three TM helices (named B, C and E) and by the buried ef helix, and capped by the cd1 and cd2 helices isolating the Q_o_ site from the intermembrane space. Additionally, ISP His161 (in bovine *bc*_1_ numbering system), which binds to one of the iron atoms in the 2Fe-2S cluster, may also contribute to the Q_o_ site when the ISP is bound to it (Esser et al. 2004).

Recent X-ray diffraction study of atovaquone complexed with cyt *bc*_1_ from *Saccharomyces cerevisiae* confirmed atovaquone is bound in the catalytic Q_o_ site, but in its ionised form (Birth et al. 2014). The oxygen from the ionised hydroxyl group interacts through hydrogen bond to the protonated His181 side chain. However, the Glu272 residue of cyt b, which provides cytb-stigmatellin complex stabilisation, is distant from atovaquone. Fifteen residues were found to stabilise the complex between atovaquone and cyt *bc*_1_ from *S. cerevisiae*, including Phe129, Tyr279 and Leu282 (Birth et al. 2014). Additionally, mutagenesis studies designed to introduce mutations associated with anti-atovaquone resistance in the homologous CYB from yeast and the bacterium *Rhodobacter capsulatus* indicated residues Phe264 (Kessl et al. 2003), Tyr268 (Kessl et al. 2005) and Leu271 (Kessl et al. 2005) in the Q_o_ site as key determinants of efficacy in ligand binding to the Pfbc_1_.

Based on the structural similarity to atovaquone, two new series of 2-hydroxy-1,4-naphthoquinones were designed as new antimalarials acting by the same mechanism of action of their template, which is to disrupt the mitochondrial electrical potential by inhibiting the cytochrome *bc*_1_ complex at the Q_o_ site (Schuck et al. 2013, Sharma et al. 2013). In these series, the alkyl side chain of atovaquone was modified while maintaining the 2-hydroxyl group, known to be important for the interaction with the Rieske ISP. Among these new atovaquone analogues, some compounds were found to have in vitro antimalarial activity with little toxicity. Moreover, there is evidence that mitochondria is the target for these compounds (Schuck et al. 2013). The aim of this work was to perform molecular modelling studies with atovaquone and antimalarial naphthoquinones on the Q_o_ site of *P. falciparum* cytochrome *bc*_1_ complex (Pfbc_1_) in order to shed some light on the structure-activity relationship of these compounds and allow us to suggest structural modifications that could improve their antimalarial activity.

## MATERIALS AND METHODS

*Homology modeling of CYB and ISP subunits of Pfbc*_*1*_ *(3D7)* - Homology modeling was carried out using the Modeller software version 9v2 using standard parameters (Sali & Blundell 1993). Amino acid sequences for the CYB and Rieske ISP subunits from *P. falciparum* (isolate 3D7) cytochrome *bc*_1_ complex were obtained from the UniprotKB/Swiss-Prot database (Swiss-Prot: Q02768, Swiss-Prot: Q8IL75). The models for CYB and ISP were built based on the crystal structures (downloaded from Protein Data Bank, PDB) of stigmatellin bound cytochrome *bc*_1_ from cow (Huang et al. 2005) (PDB: 2A06) at 2.1 Å resolution, chicken (Zhang et al. 1998) (PDB: 3H1J) at 3.0 Å resolution and yeast (Lancaster et al. 2007) (PDB: 2IBZ) at 2.3 Å resolution (Supplementary data, Fig. 1). Protein sequence alignments were performed using the T-Coffee server (Notredame et al. 2000). A set of 50 modeled structures was generated and ranked by the Modeller objective function and the top-scoring models were checked for their stereochemical and overall structural quality, using the ProCheck (Laskowski et al. 1993), Verify-3D (Eisenberg et al. 1997) and Whatcheck (Hooft et al. 1996) computer programs. A single model was selected for further analysis and as a starting structure for docking simulations.

**Fig. 1:**
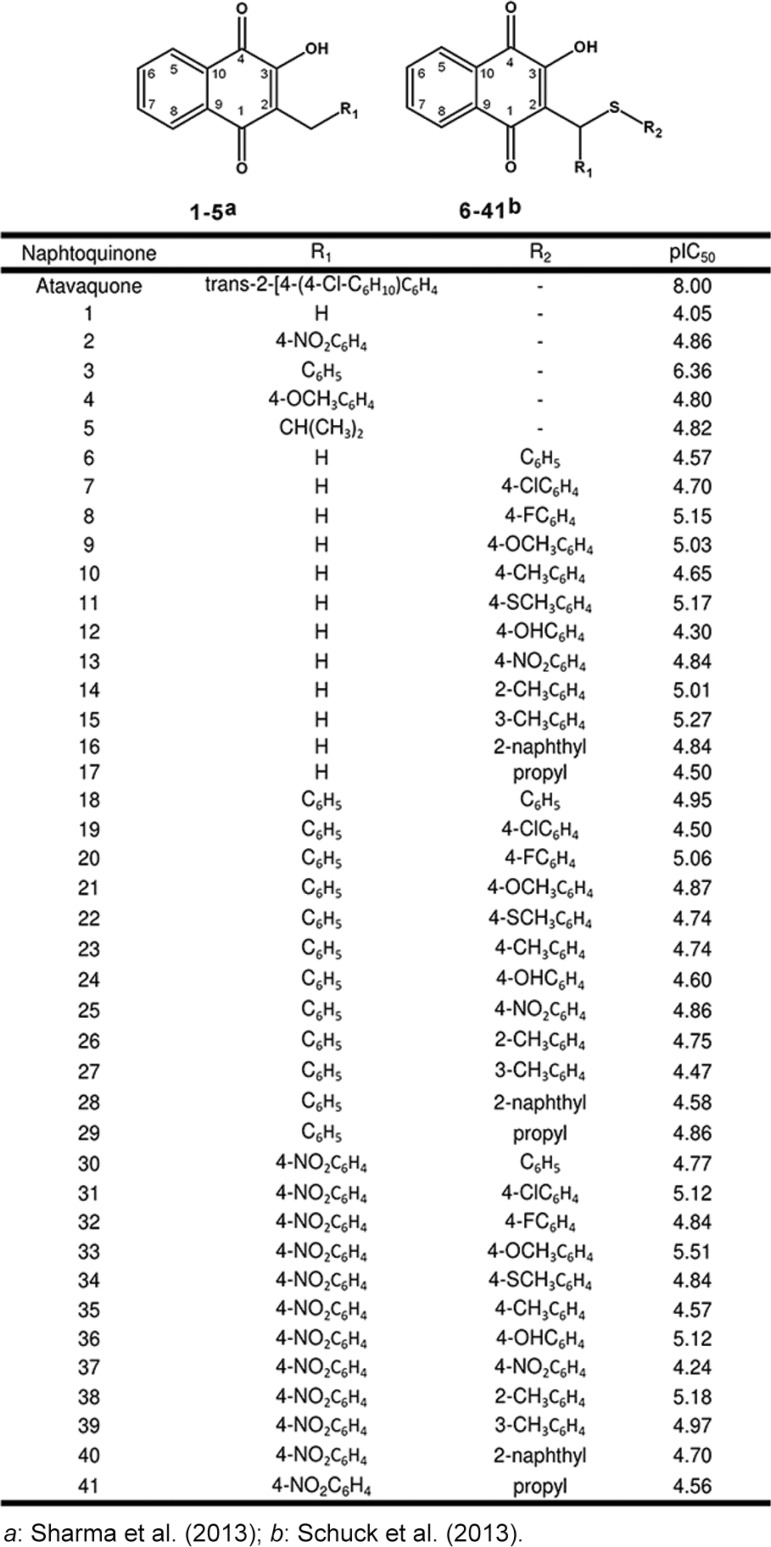
: chemical structures of atovaquone analogues and their experimental pIC50 values for antimalarial activity.

*Modeling of the Q*_*o*_ *site of Pfbc*_*1*_ *(3D7)* - For the construction of the receptor used in the docking methodology, the Pfbc_1_ subunits composing the Qo site, CYB and ISP, were superimposed on the corresponding polypeptide chains of the biologically functional assembly of the homologous yeast bc1 complex (downloaded from Protein Data Bank, PDB) (PDB: 4PD4) (Birth et al. 2014). ISP superimposition was performed such that the extrinsic domain in the *P. falciparum* protein would tightly dock on CYB (b position), as observed in the crystal structures of atovaquone bound bc_1_ complex (Birth et al. 2014). The coordinates of the 2Fe-2S cluster present in the Rieske protein were also transferred to the receptor structure due to its proximity to the Qo site pocket. The atovaquone-bound model was obtained by transferring coordinates from yeast bc_1_ PDB structure (PDB: 4PD4) (Birth et al. 2014) upon superposition on the *P. falciparum bc*_1_. The final model was obtained after addition of hydrogen atoms and running energy minimisation of all atoms through 100 steps of steepest descent energy minimisation followed by structural optimisation with conjugated gradients method until convergence was reached (gradient < 0.05 kcal/mol.A). The latter procedure was carried in Sybyl-X 1.2 modeling package (Tripos Int., St. Louis, MO, USA) using the AMBER7 FF99 force field and AMBER charges with a distance dependent dielectric constant of 80 in order to determine the solution conformations.

*Molecular docking of atovaquone and compounds 1-41 in the Q*_*o*_ *site of Pfbc*_*1*_ *(3D7)* - Structures (Fig. 1) were constructed in Spartan’10 (Wavefunction, Inc., CA, USA), with the ionised 2-hydroxyl, which was recently revealed (Birth et al. 2014). These compounds were based on the works of Schuck (molecules 1 to 5) and Sharma (molecules 6 to 41). Both works assessed the in vitro antimalarial activity employing the FACS technique by detecting the fluorescence of infected erythrocytes stained with YOYO-1 dye upon incubation for 48h with the different compounds (Schuck et al. 2013, Sharma et al. 2013).

Subsequently, all structures were optimised within the AM1 semi-empirical Hamiltonian and atomic point charges calculated by fitting the electrostatic potential. Docking was performed by Surflex-Dock (Jain 2003) v.2.51, as implemented in Sybyl-X 1.2 and AutoDock4.2 (Morris et al. 2009). In Surflex-Dock, the protomol (a representation of an idealised ligand to which putative ligands can be aligned), was derived from atovaquone bound to the Q_o_ site using the parameters threshold and bloat set to 0.50 and 0, respectively. The Surflex-Dock engine was run with the default parameters set. For running AutoDock4.2, the ligand (atovaquone) centre of mass was used as the centre of a grid calculated with 50x50x50 points and grid spacing of 0.375. Compound conformational space was explored employing the Lamarckian genetic algorithm, implemented in AutoDock4.2 (Please refer to Supplementary data, Table).

*Analysis of docking results and correlation with antimalarial activity* - Binding affinities of docked compounds were scored with Surflex-Dock scoring function and the functions implemented in CScore (Clark et al. 2002): G_SCORE - an empirical scoring function originally used in the docking program GOLD, calculated using the hydrogen bonding, complex (ligand-protein), and internal (ligand-ligand) energies; PMF_SCORE - Potential of Mean Force score calculated from a summation over all pairwise interaction terms derived from a survey of 3D structures drawn from the Protein Data Bank; D_SCORE - scoring function originally used in the docking program DOCK, calculated using only the electrostatic and van der Waals interactions between the protein and the ligand; CHEM_SCORE - an empirical scoring function calibrated to estimate binding free energies (kJ/mol), including terms for hydrogen bonding, metal-ligand interaction, lipophilic contact, and rotational entropy, along with an intercept term. Each scoring function is composed by distinct energy terms and as such may capture different aspects of the protein-ligand interaction. In the present work, Chem_score was chosen for further calculations because it rendered a better correlation between docking scores and the biological activity of the compounds.

In order to more clearly evidence the interplay of molecular target binding affinities and molecular surface properties to dictate antimalarial potency of the atovaquone analogues, we estimated the surface efficiency index (SEI) (Abad-Zapatero & Metz 2005) for these compounds from their ChemScore binding energies (denoted cSEI) and compared with pIC_50_ values. cSEI = [(-Log K_d__cs)/(PSA/100)], where K_d__cs was calculated from ChemScore binding energy using the relationship K_d_ = exp^(ΔG/RT)^. Polar surface area (PSA) and cLog p value (Log of the octanol/water partition coefficient) were calculated in Sybyl X1.2.

## RESULTS AND DISCUSSION

*Modeling of the ubiquinol oxidation (Q*_*o*_ *) site of Pfbc*_*1*_ - More than 50 X-ray structures of cytochrome *bc*_1_ complexes from different organisms are presently available at the RCSB PDB Databank (www.rcsb.org), including two from *S. cerevisiae*, either in free form (Lancaster et al. 2007) or complexed with atovaquone (Birth et al. 2014). The ideal situation for designing new *P. falciparum bc*_1_ ligands with antimalarial activity would require the crystal structure of cytochrome *bc*_1_ from the parasite but it is not available yet (Siregar et al. 2015).

**Fig. 2:**
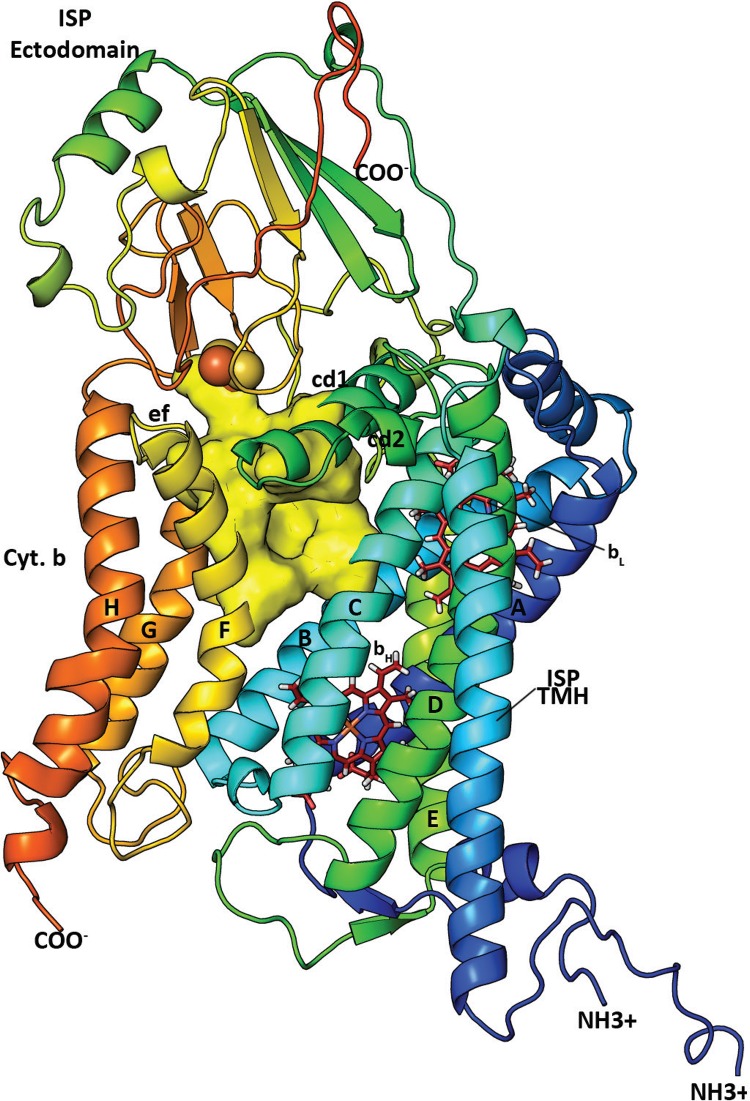
: homology model for cytochrome b (CYB) and iron-sulfur protein (ISP) subunits of *Plasmodium falciparum bc*1 complex. Cartoon representation showing: the CYB transmembrane helices (TMH, named A-H in CYB) and loop regions containing structural elements important for Qo site constitution (cd1, cd2 and ef helices); CYB low and high potential b-type hemes in sticks (labeled bL and bH, respectively); Qo pocket as a yellow channel surface; ISP TMH and ecto domain with the 2Fe-2S cluster as space-filling model (iron in orange and sulfur in yellow). The model is oriented perpendicularly to the inner mitochondrial membrane such as the intermembrane space would be at the top and the mitochondrial matrix at the bottom. Figures were made in PyMOL (www.pymol.org).

Hence, in order to generate a model for the Q_o_ site of the Pfbc_1_, we have built homology models for CYB and ISP, the relevant subunits for atovaquone and its analogues binding (Fig. 2).

The recent crystallographic structure of the yeast protein in complex with atovaquone (Birth et al. 2014) was not used as a template in the modelling of the individual CYB and ISP subunits because of it shows lower resolution (3.1 Å) than the structure from PDB code 2IBZ (2.3 Å). Instead, the yeast *bc*_1_-atovaquone complex was employed as a guide to generate the relative conformations of CYB and ISP in the Pfbc_1_. The 3D models obtained for the CYB and ISP subunits were submitted to Procheck, which indicated the presence of 98.8% and 99.3% of the residues in the most favoured or allowed regions of the Ramachadran plot, respectively (Supplementary data, Fig. 2). Scores produced by other usual protein structure validation tools were also satisfactory (Supplementary data) and inspection of major model features corroborated its validity (see below).

**Fig. 3:**
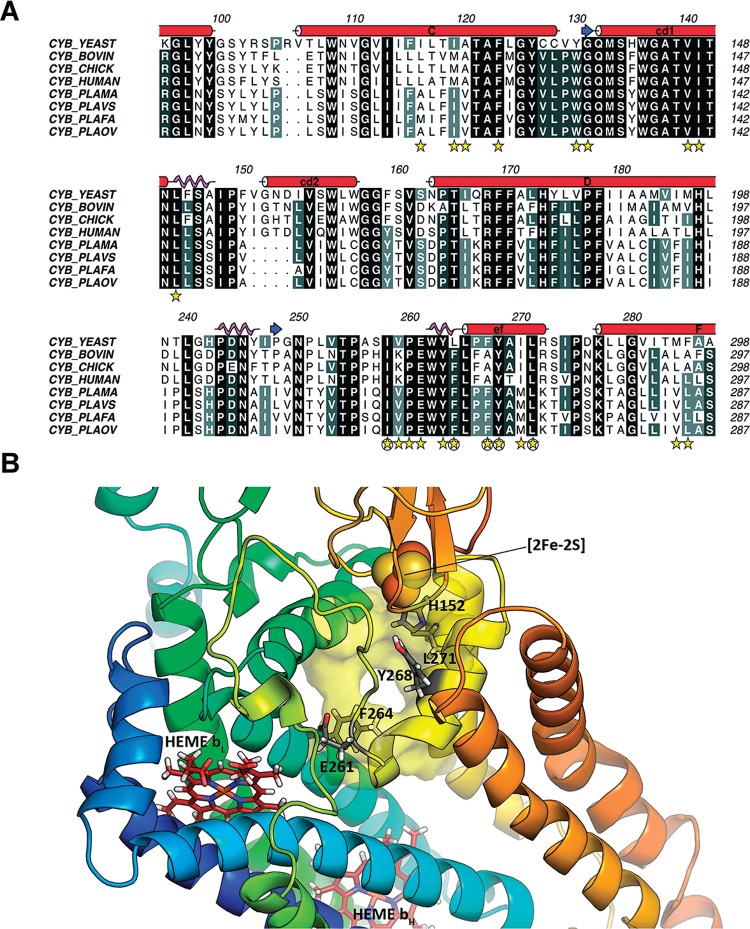
: characterisation of Qo site in *Plasmodium falciparum bc*1. (A) Alignment of cytochrome b sequences from *Plasmodium* sp causing human malaria and homologous proteins in yeast, cow, chicken and human showing structural elements composing the Qo site. Yellow stars mark residues predicted to be involved in atovaquone binding. Circumscribed stars mark residues associated with atovaquone resistance. Alignment was prepared with help of the program Aline v1.0 (Bond & Schuttelkopf 2009); (B) Zoomed view of the Qo site showing in sticks residues involved in atovaquone binding and/or resistance development.

Fig. 3 shows in detail the structural elements from the CYB subunit involved in the constitution of the Q_o_ site in cytochrome *bc*_1_. Overall, the multiple sequence alignment (panel A) shows a high level of residue conservation in helices C, cd1, cd2, D, ef and F for the four human pathogenic *Plasmodium* species (*P. malariae*, *P. vivax*, *P. falciparum* and *P. ovale*), yeast, cow, chicken and human CYB sequences. Besides, the structure obtained by the homology modelling is very similar to the 3D structure of the cytochrome *bc*_1_ from *S. cerevisiae* complexed with atovaquone (PDB code 4PD4), as indicated by the RMSD value of 0.514. However, some alignment positions containing residues predicted to be involved in atovaquone binding by *P. falciparum* CYB (see docking results below for further details) are distinct when comparing *Plasmodium sp* and vertebrate sequences (e.g., 116 and 119 in C helix; and 259, 267 and 270 in the ef loop). Those differences could be explored to design selective inhibitors for the parasite *bc*_1_ complexes.

**Fig. 4:**
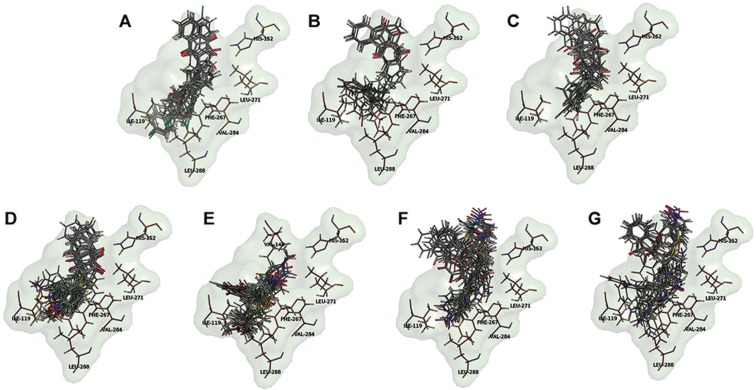
: binding mode analysis for atovaquone analogues in the Qo site in *Plasmodium falciparum bc*1. For each ligand, the top 20 scoring poses generated in Surflex-Dock are shown. (A) compound 1; (B) compound 3; (C) compound 33R; (D) compound 33S; (E) compound 37R; (F) compound 37S. For reference, selected protein residues in close contact (3.0 Å) with atovaquone are shown in sticks with carbon atoms coloured in orange (His152 from ISP and the others from CYB subunit). Ligand carbon atoms are coloured in gray. Other atoms are CPK coloured.

*Predicted binding mode of the hydroxynaphthoquinones on the Q*_*o*_ *site of Pfbc*_*1*_ - Although the anti-malarial activity of atovaquone analogues was evaluated against the *P. falciparum*, it was confirmed that atovaquone binds to the homologous yeast cyt *bc*_1_ in the catalytic Q_o_ site (Birth et al. 2014). Based on that, two different docking programs were used to explore the possible binding modes of atovaquone and its analogues in the modelled Q_o_ site of the *P. falciparum bc*_1_. Using Surflex-Dock (Jain 2003), 20 poses were generated for each docked ligand. As depicted in Fig. 4, for most ligands, the results of Surflex-Dock showed several possible binding modes. Similarly to other *bc*_1_ complexes, the Q_o_ site in *P. falciparum bc*_1_ is a long, mainly hydrophobic, V-shaped channel with an end presenting a wide “head” pocket, deep into the protein interior (close to the 2Fe-2S cluster), followed by a “neck”, where a constriction is imposed by the side chains of residues Ile141 and Leu144, culminating in a wide aperture to the lipid core of the membrane. Hence, despite the presence of any hot spots where the ligands might preferentially bind too, it was not surprising that the docked compounds, especially the smaller ones (compounds 1 and 3) could be well accommodated in many different configurations within the large search space of the Qo site.

Interestingly, many of the docked poses for atovaquone were oriented in an inverted fashion, as the naphthoquinone ring system was placed near the Q_o_ site opening. Nonetheless, such binding mode was discarded due to the lack of a strong interaction with any residue from the Rieske ISP as expected from electrochemical and spectroscopic analysis of atovaquone binding to homologous yeast *bc*_1_ (Kessl et al. 2003). Nevertheless, most poses place the C1 carbonyl of the naphthoquinone head group in hydrogen bonding distance to His152 and orient the 3-alkyl group to the neck, which is the binding mode expected from comparison with crystallographic structures for Q_o_ site inhibitors (Esser et al. 2004, Birth et al. 2014).

The results of the docking calculations with AutoDock4.2 program provided general support for data described above from Surflex-Dock calculations, in terms of the preferred poses found. Details about the AutoDock results are showed in the Supporting Information (Supplementary data, Table). All results presented in the main text are from Surflex-Dock program.

**Fig. 5:**
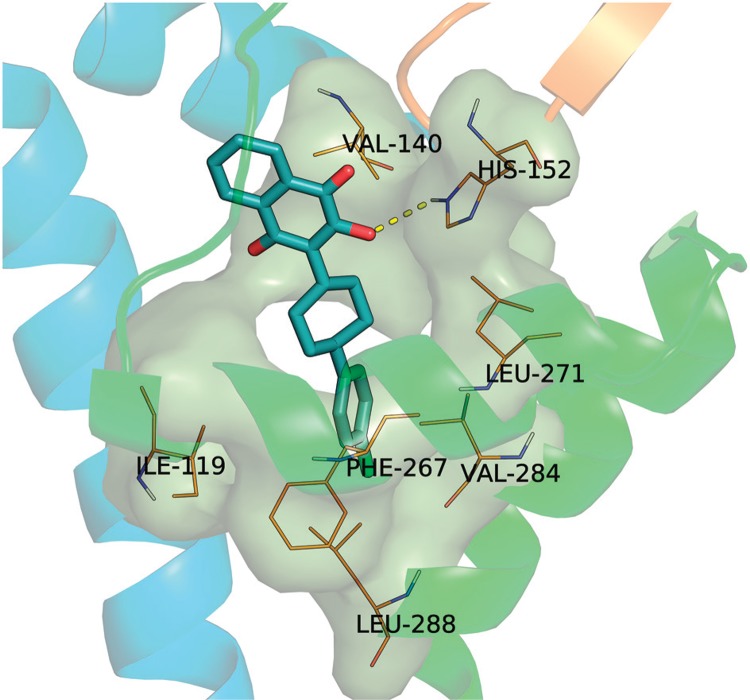
: predicted binding mode for atovaquone in the Qo site of *Plasmodium falciparum bc*1. Protein residues participating in close contacts (3.0 Å, carbons in salmon) with atovaquone (carbons in white) are show in sticks and labeled. Hydrogen bonding interaction with His152 of the Rieske ISP is shown as a dotted yellow line. The Qo site surface was rendered transparent and coloured green. Elements of secondary structure delineating the Qo site are shown in ribbon diagrams.

Fig. 5 allows a closer inspection of the proposed binding mode for atovaquone in the Q_o_ site of *P. falciparum bc*_1_. As we also consider the His152 (181 in yeast) protonated in Nε2, a hydrogen bond is established between His152 and the C2 hydroxyl oxygen atom (CO---Nε2 distance = 3.2 Å), as proposed by the X-ray structure from atovaquone bound to cyt *bc*_1_ from *S. cerevisiae* (Birth et al. 2014). Additionally, the Q_o_ site model described here has Glu261 (272 in yeast) in its ionised form with the carboxylate pointing toward the site pocket.

Atovaquone made extensive contacts, mostly van der Waals interactions, with CYB residues delineating the Q_o_ site. A π-π stacking interaction can be postulated between the aromatic rings of Tyr268 and the 2-hydroxynaphthoquinone head group. The latter is further stabilised within the pocket by the flanking side chains of Pro260 and Val140. The cyclohexyl ring of atovaquone is positioned in the neck region and predominantly contacts the aliphatic side chains of Ile141, Leu144, and Phe264. The 4-chlorophenyl ring is positioned in the channel opening and makes hydrophobic contacts with Phe267, Val 284, and Leu288. Taken as a whole, the observed contacts account for the high affinity reported for atovaquone by the *P. falciparum bc*_1_ Q_o_ site and corroborate with the binding mode observed within the crystal structure of the yeast homologous protein (Birth et al. 2014).

**Fig. 6:**
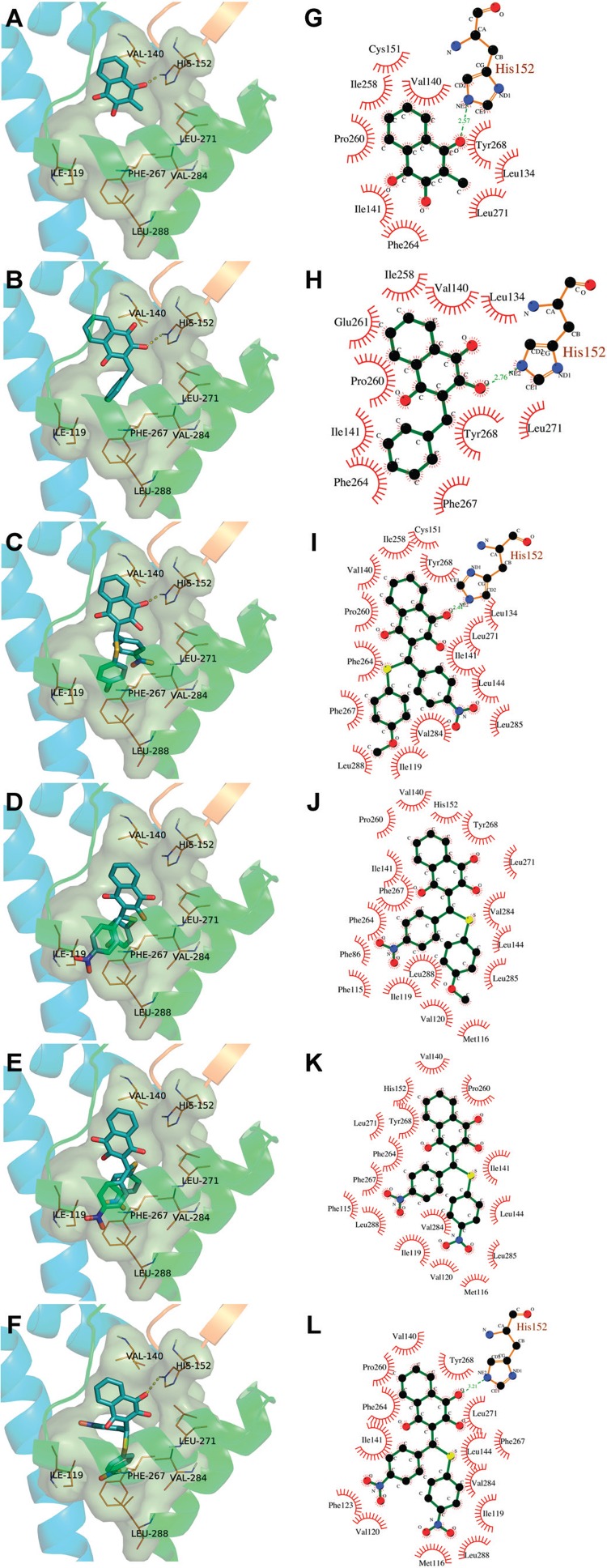
: proposed binding modes for the atovaquone analogues 1, 3, 33R, 33S, 37R, and 37S in the Qo site in *Plasmodium falciparum bc*1. Panels A-F show structural features of the binding site for compounds 1, 3, 33R, 33S, 37R, and 37S, respectively. Panels G-L show 2D representations of the main interactions between *bc*1 and compounds 1, 3, 33R, 33S, 37R, and 37S, respectively, as calculated by LigPlot+ (Laskowski & Swindells 2011). Ligands atoms numbers were omitted to aid visual inspection.

Fig. 6 presents the proposed binding modes for some of the most and the least potent compounds (in antimalarial assays) in the two 2-hydroxy-1,4-naphthoquinone series studied in this work. Analogs 3 and 33 present higher antiplasmodial activity with pIC_50_ values of 6.36 and 5.51, respectively, while compounds 1 and 37 are only moderately active with pIC_50_ values of 4.05 and 4.24, respectively. In a previous work, compounds 33 and 37 were tested as a racemic mixture (Sharma et al. 2013). Therefore, docking runs were performed with both enantiomers (R and S) aiming to recognise their individual contribution to the enzyme’s inhibition.

Similarly to the reference compound atovaquone, most analogues present a binding mode characterised by a minimum set of contacts established with His152 (Rieske ISP), Pro260 and Val140 (CYB). Ligands 3, 33R and 37S make extensive contacts involving the benzyl substituent and CYB residues Ile141, Leu144 and Phe264. A set of π-π stacking interactions were detected between Phe264 and 1, 33R and 37R/S; Phe267 and 1, 33R/S and 37R/S; and Tyr268 and 1, 3, 33R and 37R/S. Noteworthy, the binding mode of compound 1 differs from atovaquone, since it is a small compound, considering the large binding site. However, the interaction through the hydrogen bond with His152 is maintained by C4 carbonyl of the naphthoquinone derivative.

Surprisingly, for ligands 33S and 37R, docking solutions that interact through hydrogen bonds with His152 were not found. A possible explanation is the poor accommodation of the bulky R_1_/R_2_ substituents from those stereoisomers in the binding site. Conversely, for the corresponding enantiomers, docking solutions similar to the reference compound atovaquone were found. Both 33R and 37S interact via hydrogen bonding with His152, taking the C1 carbonyl oxygen atom as the acceptor. For ligand 33R, the R_2_ substituent is located at the hydrophobic pocket of the binding site, making contacts with CYB residues Leu119, Phe264, Phe267 and Leu288, which can account for the higher activity observed for compound 33. Hence, we propose that enantiomers R and S are the eutomers for compounds 33 and 37, respectively.

Overall, docking results suggest that large hydrophobic substituents, such as the phenyl groups found in compounds 3 and 33, are important for filling the deep hydrophobic Q_o_ site pocket. Compound 33 has a large non-polar R_2_ substituent, which suggests its overall higher potency to CYB. Moreover, our results indicate that the H-donor 2-hydroxyl group may not be crucial for efficient binding and inhibition of Pfbc_1_ by atovaquone analogues. The C1 carbonyl group (H-acceptor) is more often involved in the important hydrogen bonding interaction with His152.

*Correlation of ligand efficiency with antimalarial activity* - Currently, there is no single scoring function able to accurately predict binding affinities for all possible receptor-ligand systems. However, it still is a reasonable strategy to use multiple alternatives to qualitatively analyse docking results. Table I lists a set of docking scores calculated for the docking poses corresponding to the binding modes depicted in Figs 5-6. The GOLD and DOCK scoring functions (G_ and D_SCORES, respectively) are more general purpose since both were derived from force field approaches, estimating the enthalpy of binding via the pair-energy of the complex. On the other hand, those functions make no attempt to predict binding free energies once there is no entropic component in their formulation. The potential of mean force (PMF_SCORE) is a knowledge-based scoring function derived from statistical analysis of data extracted from a large number of protein-ligand complexes found in the PDB. It is intended to combine the accuracy of empirical scoring functions with the advantage of higher generality and therefore wider applicability of force-field-based scores. The other functions in Table I, i.e. Surflex and ChemScore, estimate the entropy of binding, incorporating terms for desolvation and loss of conformational flexibility and therefore attempt to predict binding free energies.

**TABLE I:** Surflex-Dock scores and cell permeability descriptors for selected docking poses of atovaquone, compounds 1, 3 and enantiomers of compounds 33 and 37

Ligand_pose	Surflex	G_ SCORE	PMF_ SCORE	D_ SCORE	CHEM_ SCORE	PSA (Å^2^)	cLogP
Atavaquone_011	0.01	-282.56	-72.03	-180.10	-47.63	76.26	5.41
1_003	4.03	-155.09	-68.47	-106.86	-31.64	96.22	1.25
3_006	4.66	-210.39	-71.78	-149.38	-39.40	80.58	2.82
33R_011	3.21	-353.01	-89.27	-211.80	-55.03	183.81	4.18
33S_000	1.72	-293.74	-66.51	-200.49	-49.45	197.37	4.18
37R_002	-2.47	-309.26	-62.34	-210.58	-50.64	212.61	3.56
37S_005	4.61	-337.24	-101.29	-202.81	-52.52	244.80	3.56

Comparison of the docking scores for binding of the atovaquone analogues at the Q_o_ site of *P. falciparum bc*_1_ reveals that compound 3, which shows the most potent antimalarial activity, does not have the highest binding affinity. In fact, according to ChemScore, analogues 33 and 37 having a 4-nitrobenzyl group attached to R1 of the naphthoquinone ring should have a more favorable binding energy than compound 3. This result is clearly not compatible with the antimalarial activities displayed by these compounds since compound 3 had an IC_50_ 10 times smaller than the compound 33. Hence, some other molecular property of these atovaquone analogues should be contributing to the observed biological activity.

Since the biological assay available here is a cellular assay, in addition to the binding affinity for the molecular target, cell membrane permeability should be accounted for in order to correctly interpret structure-activity relationships. Table I lists values calculated for two well-known molecular descriptors related to cell membrane permeability, the PSA and cLogP (Palm et al. 1996, 1997, Bergstrom et al. 2003, Abad-Zapatero & Metz 2005). cLogP values obtained in two different software’s poorly discriminated compounds according to lipophilicity, but the PSA descriptor clearly separates atovaquone and analogue3 from the rest, suggesting a superior cellular permeability for them. In particular, compounds 33 and 37 showed PSA > 140 Å^2^, which is a strong indication of poor cell membrane permeability for these compounds (Palm et al. 1996, 1997, 1997, Bergstrom et al. 2003, Abad-Zapatero & Metz 2005).

In order to correlate docking results with biological activity, ChemScore values were used for calculation of surface efficiency index (cSEI) for each ligand and further comparison of binding affinities with antimalarial potency as measured by pIC_50_ values (Table II). The cSEI showed a direct correlation with pIC_50_ (R^2^ = 0.78), i.e, the higher the cSEI values the higher is the antimalarial potency.

**TABLE II:** Comparison of predicted ligand efficiency (cSEI) with experimental pIC50 values for antimalarial activity

Naphthoquinone	pIC_50_^*a*^	cSEI^*b*^
Atovaquone	8.00	10.95
1	4.05	5.76
3	6.36	8.57
33R	5.51	5.25
33S		4.39
37R	4.24	4.17
37S		3.76

*a*: pIC_50_ = - Log IC_50_ in M concentration units; *b*: cSEI = [(-Log Kd_cs)/(PSA/100)], where Kd_cs was calculated from ChemScore binding energy using the relationship Kd = exp^(ΔG/RT)^.

It reasonably predicted the approximate activity order for atovaquone and some of most and least potent analogs having IC50 values spanning 4 orders of magnitude (atovaquone>> 3 > 1 > 33 > 37), with exception of compound 1 whose activity was overestimated. This result supports the hypothesis that, similarly to atovaquone, the Pfbc_1_ also is the molecular target for the antimalarial activity of the 2-hydroxynaphthoquinones reported here.

Based on the above results and careful visual inspection of several docked poses of the different compounds we can propose structural modifications in the 2-hydroxy-1,4-naphthoquinone nucleus that might enhance antimalarial activity. The introduction of short to medium sized side chains, especially at C7 and C8 of the naphthoquinone ring, with one or two polar groups at most, may enhance binding to the Q_o_ site while not seriously compromising the cell penetration capacity necessary for efficient antimalarial activity. The additional interactions that would be achieved with other protein residues, especially involving main chain atoms of residues, such as Ile258, or side chain atoms from residues required for efficient catalysis (e.g., Glu261), could contribute to decrease the ability of the parasite to develop drug resistance due to non-conservative amino acid mutations.

In this molecular modelling study, we analysed the interactions of 41 2-hydroxy-1,4-naphthoquinones with known antimalarial activity in the Q_o_ site of the respiratory *bc*_1_ complex from *P. falciparum*. A few representative interactions (for compounds 1, 3, 33R, 33S, 37R, and 37S) were described in depth, in order to attempt stablishing a molecular basis for their activity. Moreover, we have found a correlation between pIC50 and cSEI, an index combining predicted binding affinities from docking simulations with a calculated molecular descriptor related to cellular permeability (PSA).

Such kind of validation is of utmost importance in rational drug design since it is not rare to find unexpected binding modes after a crystal structure for the system under study is finally available.

Subsequent evaluations of analysed naphthoquinones in strains resistant to atovaquone and their use in combination with other drugs are required for a full evaluation of their potential as antimalarials. Work is underway in our group to synthesize novel atovaquone analogs with optimised antimalarial potency according to the new guidelines raised by our molecular modelling work.
