# Supplementary material for: Insights into cytochrome bc 1 complex binding mode of antimalarial 2-hydroxy-1,4-naphthoquinones through molecular modelling
Source: Mem Inst Oswaldo Cruz. 2017 Apr;112(4):299–308. doi: 10.1590/0074-02760160417 (PMC5354616; doi:10.1590/0074-02760160417)
Supplement: Supplementary file 1 [file 0074-0276-mioc-112-4-0299-suppl01.pdf]

Modeling of the ubiquinol oxidation (Q_o) site of Plasmodium falciparum (3D7) cytochrome bc_1 complex ($Pfbc_1$) - It is known that atovaquone mechanism of action is to disrupt the mitochondrial electrical potential by inhibiting the cytochrome *bc_1* complex at the Q_o site (Kessl et al. 2004). Other hydroxynaphthoquinones, such as compounds 1-41, are thought to share the same binding site. Hence, for modeling the Q_o site, we have built homology models for cytochrome b (CYB) and Rieske iron-sulfur protein (ISP), the relevant subunits of the *Plasmodium falciparum bc_1* complex (Supplementary data, Figs 1-2).

Homology modeling was carried out using the Modeller software version 9v2 (Šali & Blundell 1993). Amino acid sequences for CYB and ISP subunits from *P. falciparum* (isolate 3D7) cytochrome bc1 complex were obtained from the UniprotKB/Swiss-Prot database under accession codes Q02768 and Q8IL75, respectively. The models for CYB and ISP were built based on the crystal structures of stigmatellin bound cytochrome bc from cow (Huang et al. 2005) (PDB code 2A06, 2.1 Å resolution, 41% identity), chicken (Zhang et al. 1998) (PDB code 3H1J, 3.0 Å resolution, 45% identity) and yeast (Lancaster et al. 2007) (PDB code 2IBZ, 2.3 Å resolution, 42% identity). Protein sequence alignments were performed using the T-Coffee server (Notredame et al. 2000). A set of 50 modeled structures was generated and ranked by the objective function and the top-scoring models were checked for their and overall structural quality, using the Procheck (Laskowski et al. 1993), Verify-3D (Luthy et al. 1992), ERRAT (Colovos &Yeates 1993) and Whatcheck (Hooft et al. 1996) computer programs. A single model was selected for further analysis and as a starting structure for docking simulations.

The 3D models obtained for the CYB and ISP subunits were correctly folded as indicated by the presence of 98.8% and 99.3% of the residues in the most favored or allowed regions of the Ramachadran plot, respectively (Supplementary data, Fig. 3). Verify 3D results presented 67.21% and 53.25% of the residues from theCYBandISPsubunits respectively had an averaged 3D-1D score > 0.2; ERRAT results showed the overall quality factor of 74.7 and 63.1 for CYBandISPsubunits respectively. The models were considered useful by validation results.

For the construction of the receptor used in the docking methodology, the $Pfbc_1$ subunits composing the Q_o site, CYB and ISP, were superimposed on the corresponding polypeptide chains of the biologically functional assembly of the homologous yeast *bc_1* complex (downloaded from PDB under code 4PD4 (Birth et al. 2014)). ISP superimposition was performed such that the extrinsic domain in the *P. falciparum* protein would tightly dock on CYB (b position), as observed in the crystal structures of atovaquone bound *bc_1* complex (Birth et al. 2014). The coordinates of the 2Fe-2S cluster present in the Rieske protein were transferred to the receptor structure due to its proximity to the Q_o site pocket.

Predicted binding mode of the hydroxynaphtoquinones on the Q_o site of $Pfbc_1$ - Compounds structures were constructed in Spartan'10 (Wavefunction, Inc., CA, USA). The initial structure for atovaquone was extracted from the crystallographic complex with cyt bc_1 from *Saccharomyces cerevisiae* (PDB code 4PD4 (Birth et al. 2014)). Subsequently, all compounds structures were optimised within the AM1 semi-empirical Hamiltonian and atomic point charges calculated by fitting the electrostatic potential. For comparison purposes, docking was performed with two different programs: Surflex-Dock (Jain 2003) v.2.51, as implemented in Sybyl-X 1.2 modeling package (Tripos Int., St. Louis, MO, USA), and AutoDock4.2 (Morris et al. 2009). For Surflex-Dock, the protomol (a representation of an idealised ligand to which putative ligands will be aligned) was derived from atovaquone bound to the Q_o site using the parameters threshold and bloat set to 0.50 and 0, respectively. The atovaquone bound model was obtained by transferring coordinates from yeast *bc_1* PDB structure code 4PD4 upon superposition on the *P. falciparum bc_1*. The Surflex-Dock engine was run with the default parameters set. In addition the Surflex-Dock objective function, compounds poses were ranked for binding affinity with the scoring functions integrated (Jain 1996). For running AutoDock4, the ligand (atovaquone) centre of mass was used as the centre of a grid calculated with 50x50x50 points and grid spacing of 0.375. Compound conformational space was explored employing the Lamarckian genetic algorithm, implemented in AutoDock4.2.

The results of the docking calculations with AutoDock4.2 program provided general support for results described above for Surflex-dock calculations (Supplementary data, Fig. 3). Except for 33 and 37, docking runs resulted in configurations that predominantly grouped together in the cluster containing the best scoring solution, which was also the most populated cluster, indicating good convergence of the Lamarckian genetic algorithm. Surprisingly, all solutions generated by AutoDock for compound 1 is oriented to interact with Glu261, instead of His152. However, as discussed, this compound is very small when compared with the binding site and can assume differrent orientations inside the Q_o site in *P. falciparum bc_1*.

Compounds 33 and 37 can also assume different orientations inside the Q_o site, specially the S enantiomers since some poses presented the polar group (i.e., the nitrobenzyl) oriented to the Hist152. However, this orientation is not favored by others interactions inside the active site.

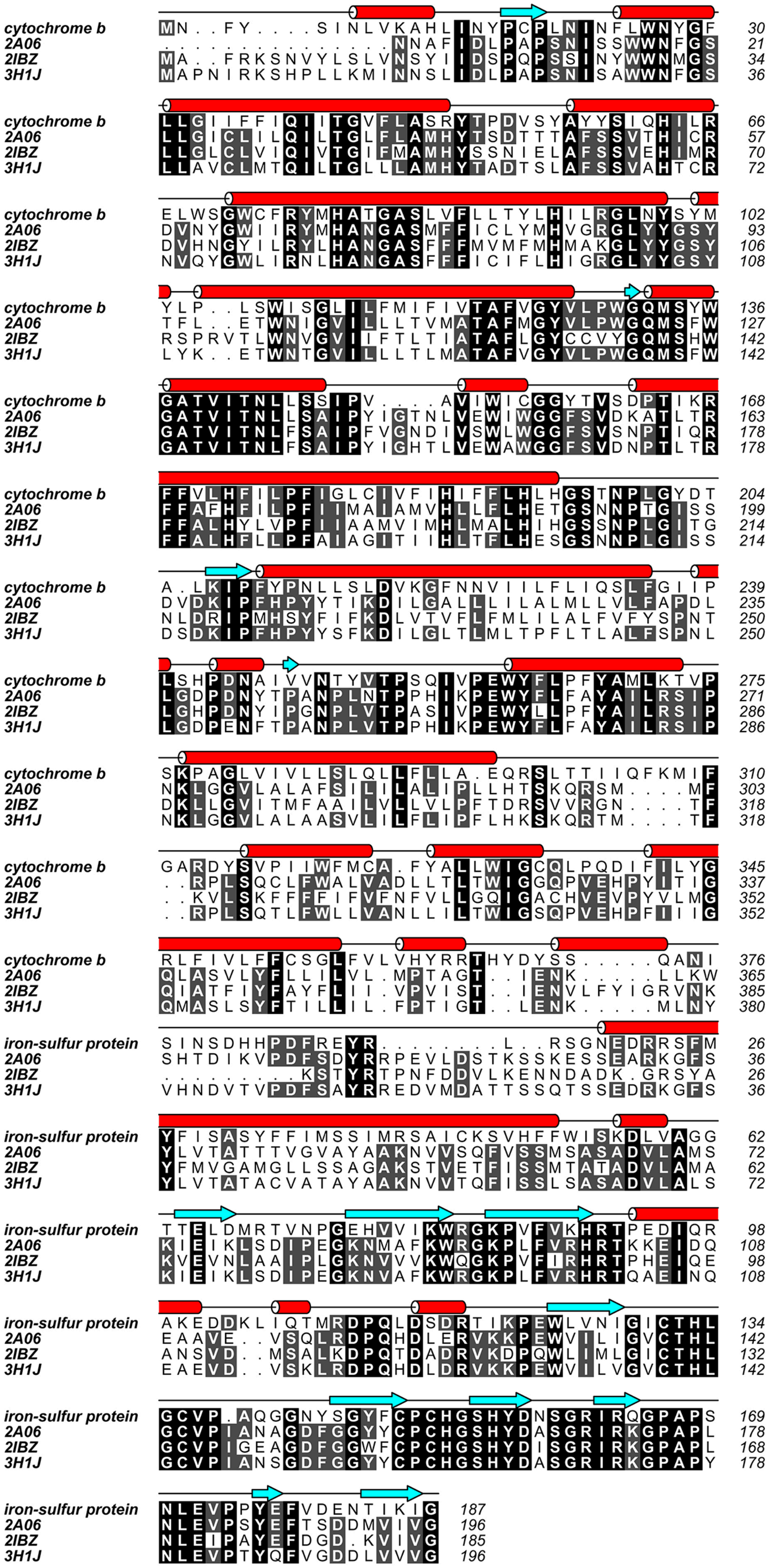

Fig. 1: alignment of cytochrome b and iron-sulfur protein subunits and the templates (PDB code 2A06, 2IBZ and 3H1J). Secondary structures of the model were obtained by DSSP program [11]. The identical residues are shadowed in black while conserved residues (75%) are in gray.

Supplementary data

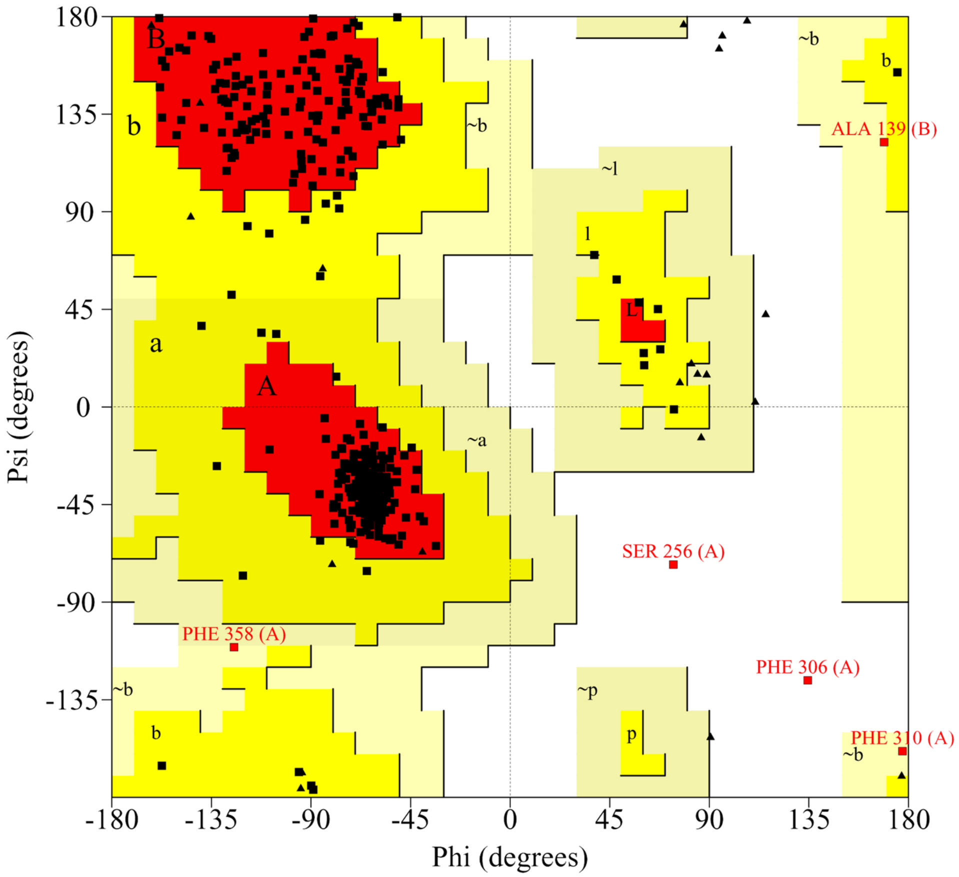

Plot statistics

Residues in most favoured regions [A,B,L]	453	92.3%
Residues in additional allowed regions [a,b,l,p]	33	6.7%
Residues in generously allowed regions [~a,~b,~l,~p]	3	0.6%
Residues in disallowed regions	2	0.4%
	----	------
Number of non-glycine and non-proline residues	491	100.0%
Number of end-residues (excl. Gly and Pro)	7	
Number of glycine residues (shown as triangles)	35	
Number of proline residues	32	

Total number of residues	565	

Fig. 2: ramachandran plot of the model built from cytochrome b (chain A) and iron sulfur protein (chain B) subunits.

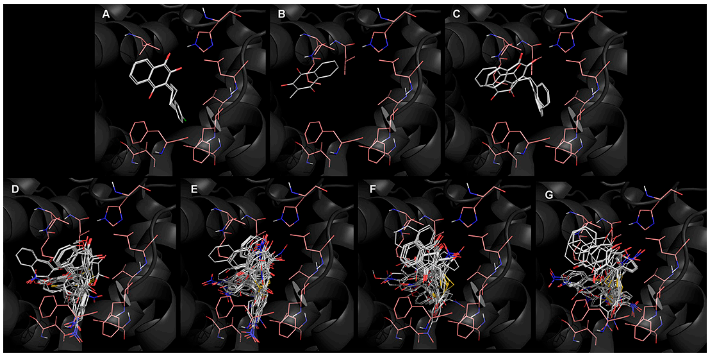

Fig. 3: binding mode analysis for atovaquone analogues in the Q_o site in *P. falciparum bc*$_1$. For each ligand, the top 20 scoring poses generated in AutoDock4.2 are shown. (A) Atavaquone; (B) compound 1; (C) compound 3; (D) compound 33R; (E) compound 33S; (E) compound 37R; (F) compound 37S. For reference, selected protein residues in close contact (3.0 Å) with atovaquone are shown in sticks with carbon atoms colored in salmon (His152 from ISP and the others from CYB subunit). Ligand carbon atoms are colored in white. Other atoms are CPK-colored. Hydrogen bonds are indicated by yellow dotted lines.

TABLE

Results of AutoDock[a] for atovaquone derivatives on the Q_o site of *Plasmodium falciparum bc*$_1$ complex

	Atovaquone	1	3	33R	33S	37R	37S
Number of clusters	2	1	1	14	18	17	15
Number of conformations in the most populated cluster	47	50	50	11	8	9	10
Estimated free energy binding (kcal/mol)[b]	-11.21	-7.07	-9.46	-10.28	-10.34	-10.60	-9.99

a: the program AutoDockTools (ADT) was used to prepare the protein and the ligand. Gasteiger charges were assigned and nonpolar hydrogen atoms were merged. The grid maps of docking studies were computed using the AutoGrid4. Center of the ligand was used as the center of the grid and it was calculated with 50x50x50 points with grid spacing of 0.375 were calculated. Docking studies were carried out using the program AutoDock4 [13]. Compound conformational space was explored employing the Lamarckian genetic algorithm. The parameters for LGA were defined as follows: a maximum number of 250,000 energy evaluations; mutation and crossover rates of 0.02 and 0.8, respectively. The number of docking runs was set to 50. After docking, all structures generated were assigned to clusters based on a tolerance of 2.0 Å all-atom RMSD from the lowest-energy structure; *b*: conformations of the lowest docked energy of best scoring cluster.
